# Subcellular spatial resolution achieved for deep-brain imaging in vivo using a minimally invasive multimode fiber

**DOI:** 10.1038/s41377-018-0111-0

**Published:** 2018-12-19

**Authors:** Sebastian A. Vasquez-Lopez, Raphaël Turcotte, Vadim Koren, Martin Plöschner, Zahid Padamsey, Martin J. Booth, Tomáš Čižmár, Nigel J. Emptage

**Affiliations:** 1 Department of Pharmacology, University of Oxford, Mansfield Road, Oxford, OX1 3QT UK; 2 Department of Engineering Science, University of Oxford, Parks Road, Oxford, OX1 3PJ UK; 3 School of Engineering, Physics and Mathematics, College of Art, Science & Engineering, University of Dundee, Nethergate, Dundee, DD1 4HN Scotland UK; 4 Institute of Scientific Instruments of the CAS, Královopolská 147, 612 64 Brno, Czech Republic

## Abstract

Achieving intravital optical imaging with diffraction-limited spatial resolution of deep-brain structures represents an important step toward the goal of understanding the mammalian central nervous system^1–4^. Advances in wavefront-shaping methods and computational power have recently allowed for a novel approach to high-resolution imaging, utilizing deterministic light propagation through optically complex media and, of particular importance for this work, multimode optical fibers (MMFs)^5–7^. We report a compact and highly optimized approach for minimally invasive in vivo brain imaging applications. The volume of tissue lesion was reduced by more than 100-fold, while preserving diffraction-limited imaging performance utilizing wavefront control of light propagation through a single 50-μm-core MMF. Here, we demonstrated high-resolution fluorescence imaging of subcellular neuronal structures, dendrites and synaptic specializations, in deep-brain regions of living mice, as well as monitored stimulus-driven functional Ca^2+^ responses. These results represent a major breakthrough in the compromise between high-resolution imaging and tissue damage, heralding new possibilities for deep-brain imaging in vivo.

Presently, non-invasive (surface) high-resolution imaging of brain tissue can achieve micrometer resolution up to penetration depths of ~1 mm^1^. Beyond this limit, scattering and optical aberrations introduced by the heterogenous refractive index distribution within brain tissue prohibit observing subcortical structures (even in mice), many of which are implicated in important neuronal processes such as memory formation and gating of sensory and motor information, as well as neurological diseases^1–4^. The importance of visualizing these brain regions has precipitated the development of diverse optical strategies, including the removal of overlying cortical structures^8^ and insertion of fiber bundles^9^ and graded index (GRIN) lenses^10,11^. Unfortunately, these approaches create substantial mechanical lesions of the tissue, precipitating neuropathological responses that include inflammation and gliosis^12^ and possibly ultimately compromising the physiology of neuronal networks and behavior of the animal^13,14^. Here, we adopted an endoscopic approach utilizing multimode optical fibers (MMFs). The principles behind the MMF imaging method are detailed in the Supplementary Methods^5–7^. The optical geometry was optimized to provide the functional stability and mobility necessary for use in vivo (Fig. 1a). Integral to the system is a liquid-crystal spatial light modulator (LC-SLM), which enabled manipulation of the propagating light field through the optical path comprising an arbitrary MMF length. Prior to the commencement of imaging, the LC-SLM was used in a calibration procedure during which we acquired a transmission matrix (TM), fully describing the light field propagation within the optical system^15^. The availability of the system-specific TM then allowed us to produce a set of field modulations, which were employed in the image acquisition procedure. Each of these modulations, when applied at the LC-SLM, produced a diffraction-limited spot at a specific location across the fiber output plane. Importantly, spots may be generated at an arbitrary distance from the distal fiber facet.

**Fig. 1:**
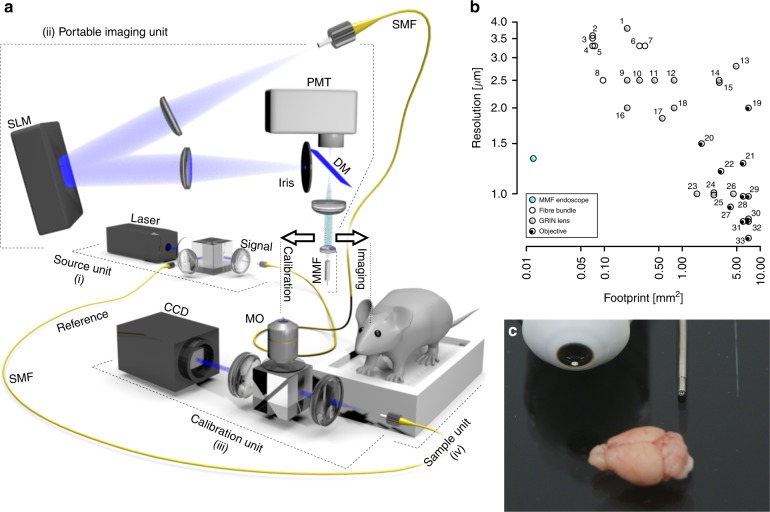
MMF imaging system for minimally invasive deep-brain in vivo imaging achieves diffraction-limited spatial resolution. **a** Schematic of the experimental system. (i) The source unit distributes the laser light for fluorescence excitation and calibration. (ii) The portable imaging arm controls the light propagation through the MMF by wavefront shaping using an LC-SLM. (iii) The calibration unit is used for the acquisition of the transmission matrix prior to imaging. (iv) The sample unit holds tissue sections and head-fixed mice. DM dichroic mirror, MMF multimode fiber, MO microscope objective, PMT photomultiplier tube, SMF single-mode fiber. **b** Relationship between image resolution and instrument footprint for in vivo light-based imaging modalities (Supplementary Table S1). The MMF data point is shown in blue. **c** Picture of a mouse brain (bottom) with optical components for in vivo imaging (top): a 60× water immersion objective (left), a GRIN lens (middle), and the 50-μm-core MMF used in this study (right)

The source unit of the system distributes laser light (488 nm) into two single-mode optical fibers, one to deliver the excitation signal to the LC-SLM and the other to provide a reference signal during calibration. The main optical arm, comprising the LC-SLM, MMF probe, relay optics, and a fluorescence detection unit, was designed to be compact and is embedded within a robust caged framework housed on a three-dimensional micro-positioning stage to facilitate alignment during calibration as well as navigation of the MMF probe into the brain tissue (Supplementary Methods). Finally, the calibration arm, used only during TM acquisition, relays the MMF probe output signal to a camera, where it interferes with the reference beam. The implementation of a GPU-accelerated toolbox for LC-SLM control^7^ enabled the acquisition of the full transformation matrix of a 50-μm-core MMF in <4 min. Imaging can be performed immediately after the TM acquisition, and the calibration did not change in time, provided that the fiber was not deformed and that its position relative to the rest of the optical system was not changed (Supplementary Note S1). This combination of procedures provides the basis for fiber-based volumetric point-scanning fluorescence microendoscopy, with an endoscopic probe whose diameter is several fold smaller than any used previously. Diffraction-limited performance was achieved (FWHM of the excitation PSF: 1.27 ± 0.01 μm laterally and 20.2 ± 1.4 µm axially; resolution of fluorescent objects from the Rayleigh criterion: 1.35 μm; both measurements at 488 nm; NA: 0.22; Supplementary Fig. S1a, b) and, importantly, was maintained up to 100 µm from the distal facet (Supplementary Fig. S1c)—i.e., throughout the range of dynamic refocusing used in this study. This represents an enormous step in the trade-off between image resolution and device footprint (Fig. 1b, c and Supplementary Table S1).

To assess the performance of our system when imaging neuronal structures, we first conducted imaging trials in ex vivo brain slices from the rat hippocampus (Fig. 2). Fluorescently labeled neurons were imaged using both a standard confocal microscope equipped with a 60× water immersion objective (Fig. 2b, c green images) and our fiber-based system (Fig. 2b, c gray images). Dendritic spines and axonal boutons were clearly visible, with identical structures identifiable in both MMF and confocal images. These results suggest that the device would be suitable for structural imaging studies. We wish to stress that our digital scanning approach is free from the granulous artifacts commonly observed when using computational approaches^16,17^ because these can be easily confused with synaptic structures. This is a particular concern where high-resolution images are collected from sparsely labeled samples over a small area, as it is frequently the case in neurobiology^18,19^. Furthermore, previous implementations of the MMF imaging system have failed at achieving sufficient spatial resolution for the visualization of dendritic spines and exclusively reported images of soma^12,20^. Our results offer robust validation of the MMF approach for the acquisition of fluorescent images in living tissue. A limitation is that the field-of-view currently corresponds to the core diameter of the MMF (50 μm); if necessary, a wider field-of-view can be achieved using an MMF with a larger core diameter (105 μm) while maintaining the spatial resolution (Supplementary Fig. S2). Using MMF with a much larger diameter would increase invasiveness but may also be challenging to implement because it would require a wavefront-shaping device with many pixels because the number of input modes required scales to the square of the core diameter.

**Fig. 2:**
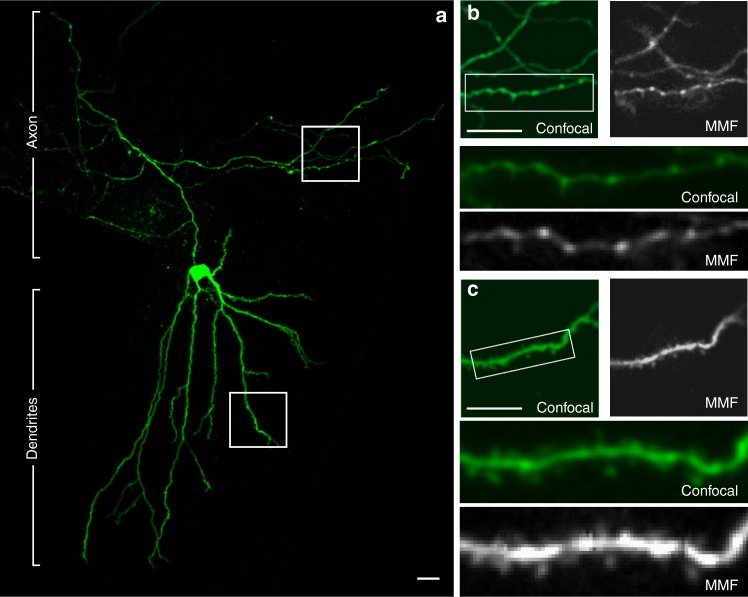
MMF imaging of ex vivo tissue reveals dendritic spines and axonal boutons. **a** Confocal image of a hippocampal neuron in a rat brain slice. **b**, **c** Structural images through the MMF (**b**, **c** grey images) and confocal microscopy (**b**, **c** green images) of the same regions of the neuron in (**a**). Axonal boutons (**b**) and dendritic spines (**c**) were clearly identified using each imaging modality. Scale bars: 20 μm

Having demonstrated that the system performs well when imaging living neuronal tissue ex vivo, we set out to explore whether we could achieve comparable results in vivo. Here we sought to image neurons from deep regions of the intact brain of live mice. We used transgenic Thy1-GFP line M mice that express fluorescently labeled neurons sparsely throughout the nervous system, an approach commonly used for in vivo neuronal structural imaging studies (Supplementary Note S2)^18^. Because the diameter of our fiber was small, 125 μm inclusive of cladding, we could insert the fiber directly into the brain tissue via a small craniotomy and image in real-time as we advanced slowly through the tissue. This is a significant improvement over existing methods for deep-brain imaging with equivalent spatial resolution that require extensive surgery and aspiration of the overlying brain tissue. On identifying our target structure, we could begin imaging immediately, again in marked contrast with other endoscopic brain imaging methods that require several days post-surgery before imaging can commence^12^. Critically, we saw little evidence of damage to blood vessels; thus, images were not obscured by tissue bleeding. Although chronic imaging with the MMF system was not demonstrated here, its minimal invasiveness show promise in minimizing the post-implantation recovering period.

Figure 3 shows images of a fluorescent neuron in the dorsal striatum imaged after lowering a fiber 1.8 mm into the brain of an anesthetized mouse and collecting an image stack at different focal planes beneath the fiber (Fig. 3a, b). A dendritic branch on which there were dendritic spines was clearly identifiable (Fig. 3c) and the best in-focus axial plane identified using digital refocusing. The ability to adjust the focal plane while maintaining the fiber at a fixed position represents a further considerable advantage of the MMF fiber system for in vivo imaging. The imaging plane can be adjusted over a range of 0–100 μm from the fiber facet with no movement of the fiber and therefore no mechanical consequence for the brain tissue. This further minimizes the impact of fiber placement into the tissue. The extent to which the placement of the fiber impacts upon the neuronal tissue during image collection is shown in a post-mortem section of brain tissue (Fig. 3d). Labeled neurons and their dendritic processes remained alive and intact even when located in close proximity to the fiber tract (Fig. 3d, inset).

**Fig. 3:**
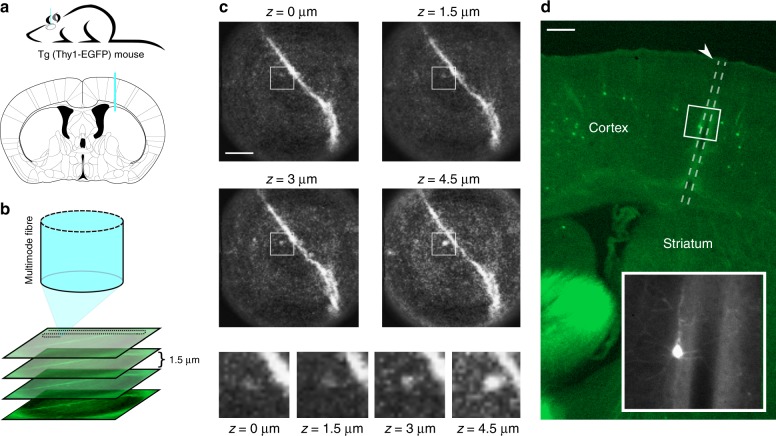
MMF imaging system achieves in vivo visualization of dendrites and their spines in the dorsal striatum of mice. **a** Imaging was performed by lowering an MMF 1.8 mm into the brain of an anesthetized Thy1-GFP line M mouse to reach the dorsal striatum (top). Atlas depiction of the region of the striatum imaged in (**c**) adapted from the Allen Mouse Brain Atlas (bottom) with fiber placement in blue. **b** Fluorescence imaging was performed at multiple distances (depths, reference plane: 50 μm) from the distal facet of the fiber by calibrating the system to different focal planes. **c** Dendritic spines were clearly identifiable, and their three-dimensional structure became visible when varying the focal plane. Scale bar: 10 μm. **d** Post-mortem histological section of the mouse brain imaged in (**c**) showing the path of the fiber through the cortex. Scale bar: 200 μm. The inset shows that the structure of cortical neurons is preserved even around the margins of the fiber track

While structural imaging is a valuable technique, we sought to assess whether fiber-based dynamic imaging can also be achieved. Our system detected changes in fluorescence elicited in a neuron loaded with the Ca^2+^ indicator OGB-1, in ex vivo brain slices from the rat hippocampus, after exposure to potassium (45 mM; Fig. 4a, b). The variation in fluorescence (dF/F) was calculated by subtracting the whole image taken immediately after the addition of potassium (Fig. 4b) from the one taken immediately before (Fig. 4a). For in vivo imaging, neurons of the medial geniculate body (MGB)—a part of the auditory thalamus—of C57BL/6 mice were sparsely labeled with the Ca^2+^ reporter GCaMP6m in order to record action potentials elicited in response to auditory stimuli. We exploited the digital point-scanning capability of our SLM-based system for random-access imaging of a reduced number of pixels at 33.3 Hz. Using this approach, we could reliably observe large stimulus-driven Ca^2+^ transients from sound-responsive neurons in anesthetized mice in vivo (Fig. 4b–d). In its current configuration, the imaging system can operate at a scan rate of ~10 ms per pixel, determined by the maximum refresh rate of 100 Hz for the LC-SLM (frame rate = 2.4 s per 120 × 120-pixel frame). The LC-SLM could be combined with an acousto-optic deflector to increase the frame rate^6,21^, but this comes at the cost of increasing the system complexity. Digital micro-mirror devices are an alternative to LC-SLMs for spatial-light modulation^22–24^, achieving update-rate orders of magnitude larger than LC-SLM (up to 22 kHz), and could be incorporated into our geometry.

**Fig. 4:**
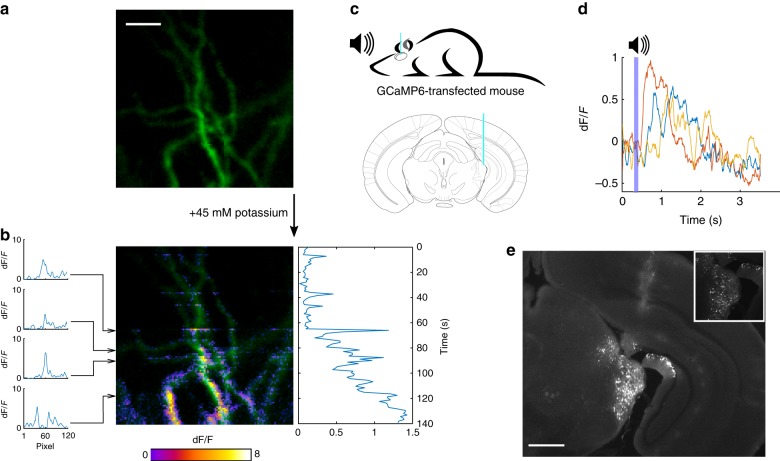
The MMF-based system captures dynamic changes in neuronal Ca^2+^ signals. **a** A neuron in a rat organotypic hippocampal slice was filled with the Ca^2 + ^-sensitive dye OGB-1. **b** After bath application of 45 mM potassium, a gradual increase in intracellular Ca^2+^ resulting from the depolarization of the neuron was detected through dynamic changes in fluorescence. The traces on the left show the variation in florescence during the scanning of individual lines of the image, each 120 pixels. The trace on the right is the average dF/F for all scanned lines and shows the gradual increase in intracellular Ca^2+^ from the moment in which potassium is added to the bath. Scale bar: 10 μm. **c** An MMF was lowered 3 mm into the MGB of an anesthetized mouse presented with auditive stimuli (top). Atlas depiction indicating the placement of the MMF (blue); adapted from the Allen Mouse Brain Atlas (bottom). **d** Calcium responses recorded from a single pixel were elicited by repeated presentation of a 100-ms pure tone of 16 kHz (purple bar). Color traces show the responses from individual trials. **e** Post-mortem histological analysis showed sparse expression of the genetically encoded calcium indicator GCaMP6m in the MGB and fiber track. The inset shows the MGB neuron labeling. Scale bar: 1 mm

In summary, we have achieved structural and functional in vivo fluorescent imaging of neurons within deep-brain structures of mice that we believe to be the least invasive, deep-brain, high-resolution approach reported to date. This method provides a route to achieving high-resolution optical access to deep-brain subcellular processes in living and ultimately in freely behaving animals, one of the most unique and appealing possibilities of MMF imaging systems, with minimal disruption to the associated circuitry. This study also prominently demonstrates the applications of wavefront-shaping microscopy in biomedical research^25^ and potential future advances in minimally invasive imaging in vivo in a multitude of organs. Future developments will now aim to achieve the scanning speeds necessary for dynamic imaging over wider areas, enabling neuronal population activity to be monitored and a critical step toward brain imaging in freely moving animals (Supplementary Note S3). Additionally, optical sectioning strategies must be devised for the imaging to be truly three-dimensional. Some progress has been made in this regard by implementing a form of holographic confocal microscopy with reflectance contrast^26^. More suitable for fluorescence imaging would be to exploit the intrinsic optical sectioning offered by two-photon microscopy, which has been demonstrated through a step-index MMF^27^. Finally, super-resolution imaging could be achieved by implementing a STED configuration because, unlike structured illumination or localization microscopy, the spectrally broad fluorescence would not have to be imaged through the MMF—i.e., the control of two monochromatic illumination beams would be sufficient^28^.

## Supplementary information

Supplementary material
