# Supplementary material for: Subcellular spatial resolution achieved for deep-brain imaging in vivo using a minimally invasive multimode fiber
Source: Light Sci Appl. 2018 Dec 19;7:110. doi: 10.1038/s41377-018-0111-0 (PMC6298975; doi:10.1038/s41377-018-0111-0)
Supplement: Supplementary file 1 — Supplementary material [file 41377_2018_111_MOESM1_ESM.docx]

**Supplementary Information**

**Subcellular spatial resolution achieved for deep-brain imaging *in vivo* using a minimally invasive multimode fiber**

Sebastian A. Vasquez-Lopez^1,5^, Raphaël Turcotte^1,2,5^, Vadim Koren^1,5^, Martin Plöschner^3^, Zahid Padamsey^1^, Martin J. Booth^2^, Tomáš Čižmár^3,4^ and Nigel J. Emptage^1^*.

1. Department of Pharmacology, University of Oxford, Mansfield Road, Oxford OX1 3QT, United Kingdom

2. Department of Engineering Science, University of Oxford, Parks Road, Oxford OX1 3PJ, United Kingdom

3. School of Engineering, Physics and Mathematics, College of Art, Science & Engineering, University of Dundee, Nethergate, Dundee DD1 4HN, Scotland, United Kingdom

4. Institute of Scientific Instruments of the CAS, Královopolská 147, 612 64 Brno, Czech Republic

5. These authors contributed equally to this work. ***Corresponding author**: nigel.emptage@pharm.ox.ac.uk

**Supplementary Figures**

**Figure S1**. Focusing at the distal facet of a 50 μm-core MMF. (a) High dynamic range image of the focused excitation light. Scale bar: 20 μm. (b) Average profile of the excitation light at the center of the MMF (n = 5). (c) Normalized lateral full width at half maximum (FWHM) of the focused excitation light as a function of the distance from the distal facet of the MMF (n = 3). The normalization was made to the average FWHM value in the range of 0 to 90 µm.

**Figure S2**. Focusing at the distal facet of a 105 μm-core MMF. (a) High dynamic range image of the focused excitation light (488 nm) at the center of the MMF. Scale bar: 20 μm. (b) Average intensity profile of the excitation at the center of the MMF (n = 3). (c) High dynamic range image of the focused excitation light (488 nm) at the edge of the MMF. Scale bar: 20 μm. (d) Average intensity profile of the excitation at the edge of the MMF (n = 3).

**Supplementary Table S1.** List of studies, included as data points in Figure 1b, which implement various forms of *in vivo* light-based brain imaging.

| **#** | **Reference** |
| --- | --- |
|  | **Fiber bundle** |
| 2 | Goto, A., Nakahara, I., Yamaguchi, T., Kamioka, Y., Sumiyama, K., Matsuda, M., ... & Funabiki, K. (2015). Circuit-dependent striatal PKA and ERK signaling underlies rapid behavioral shift in mating reaction of male mice. *Proceedings of the National Academy of Sciences*, *112*(21), 6718-6723. |
| 3 | Yamaguchi, T., Goto, A., Nakahara, I., Yawata, S., Hikida, T., Matsuda, M., ... & Nakanishi, S. (2015). Role of PKA signaling in D2 receptor-expressing neurons in the core of the nucleus accumbens in aversive learning. *Proceedings of the National Academy of Sciences*, *112*(36), 11383-11388. |
| 4 | Chen, X., Cao, H., Saraf, A., Zweifel, L. S., & Storm, D. R. (2015). Overexpression of the type 1 adenylyl cyclase in the forebrain leads to deficits of behavioral inhibition. *Journal of Neuroscience*, *35*(1), 339-351. |
| 5 | Soden, M. E., Jones, G. L., Sanford, C. A., Chung, A. S., Güler, A. D., Chavkin, C., ... & Zweifel, L. S. (2013). Disruption of dopamine neuron activity pattern regulation through selective expression of a human KCNN3 mutation. *Neuron*, *80*(4), 997-1009. |
| 6 | Szabo, V., Ventalon, C., De Sars, V., Bradley, J., & Emiliani, V. (2014). Spatially selective holographic photoactivation and functional fluorescence imaging in freely behaving mice with a fiberscope. *Neuron*, *84*(6), 1157-1169. |
| 7 | Vincent, P., Maskos, U., Charvet, I., Bourgeais, L., Stoppini, L., Leresche, N., ... & Paupardin‐Tritsch, D. (2006). Live imaging of neural structure and function by fibred fluorescence microscopy. *EMBO reports*, *7*(11), 1154-1161. |
| 8 | Bharali, D. J., Klejbor, I., Stachowiak, E. K., Dutta, P., Roy, I., Kaur, N., ... & Stachowiak, M. K. (2005). Organically modified silica nanoparticles: a nonviral vector for in vivo gene delivery and expression in the brain. *Proceedings of the National Academy of Sciences of the United States of America*, *102*(32), 11539-11544. |
|  |  |
|  | **GRIN lens** |
| 1 | Murayama, M., Pérez-Garci, E., Nevian, T., Bock, T., Senn, W., & Larkum, M. E. (2009). Dendritic encoding of sensory stimuli controlled by deep cortical interneurons. *Nature*, *457*(7233), 1137. |
| 9 | Cox, J., Pinto, L., & Dan, Y. (2016). Calcium imaging of sleep–wake related neuronal activity in the dorsal pons. *Nature communications*, *7*, 10763. |
| 10 | Betley, J. N., Xu, S., Cao, Z. F. H., Gong, R., Magnus, C. J., Yu, Y., & Sternson, S. M. (2015). Neurons for hunger and thirst transmit a negative-valence teaching signal. *Nature*, *521*(7551), 180. |
| 11 | Kitamura, T., Sun, C., Martin, J., Kitch, L. J., Schnitzer, M. J., & Tonegawa, S. (2015). Entorhinal cortical ocean cells encode specific contexts and drive context-specific fear memory. *Neuron*, *87*(6), 1317-1331. |
| 12 | Sun, C., Kitamura, T., Yamamoto, J., Martin, J., Pignatelli, M., Kitch, L. J., ... & Tonegawa, S. (2015). Distinct speed dependence of entorhinal island and ocean cells, including respective grid cells. *Proceedings of the National Academy of Sciences*, *112*(30), 9466-9471. |
| 13 | Flusberg, B. A., Nimmerjahn, A., Cocker, E. D., Mukamel, E. A., Barretto, R. P., Ko, T. H., ... & Schnitzer, M. J. (2008). High-speed, miniaturized fluorescence microscopy in freely moving mice. *Nature methods*, *5*(11), 935. |
| 14 | Ghosh, K. K., Burns, L. D., Cocker, E. D., Nimmerjahn, A., Ziv, Y., El Gamal, A., & Schnitzer, M. J. (2011). Miniaturized integration of a fluorescence microscope. *Nature methods*, *8*(10), 871. |
| 15 | Ziv, Y., Burns, L. D., Cocker, E. D., Hamel, E. O., Ghosh, K. K., Kitch, L. J., ... & Schnitzer, M. J. (2013). Long-term dynamics of CA1 hippocampal place codes. *Nature neuroscience*, *16*(3), 264. |
| 16 | Jennings, J. H., Ung, R. L., Resendez, S. L., Stamatakis, A. M., Taylor, J. G., Huang, J., ... & Ramakrishnan, C. (2015). Visualizing hypothalamic network dynamics for appetitive and consummatory behaviors. *Cell*, *160*(3), 516-527. |
| 18 | Pinto, L., & Dan, Y. (2015). Cell-type-specific activity in prefrontal cortex during goal-directed behavior. *Neuron*, *87*(2), 437-450. |
| 23 | Barretto, R. P., Messerschmidt, B., & Schnitzer, M. J. (2009). In vivo fluorescence imaging with high-resolution microlenses. *Nature methods*, *6*(7), 511. |
| 17, 24 | Barretto, R. P., Ko, T. H., Jung, J. C., Wang, T. J., Capps, G., Waters, A. C., ... & Schnitzer, M. J. (2011). Time-lapse imaging of disease progression in deep brain areas using fluorescence microendoscopy. *Nature medicine*, *17*(2), 223. |
| 25, 26 | Attardo, A., Fitzgerald, J. E., & Schnitzer, M. J. (2015). Impermanence of dendritic spines in live adult CA1 hippocampus. *Nature*, *523*(7562), 592. |
|  |  |
|  | **Objective** |
| 19 | Villette, V., Malvache, A., Tressard, T., Dupuy, N., & Cossart, R. (2015). Internally recurring hippocampal sequences as a population template of spatiotemporal information. *Neuron*, *88*(2), 357-366. |
| 20 | Danielson, N. B., Kaifosh, P., Zaremba, J. D., Lovett-Barron, M., Tsai, J., Denny, C. A., ... & Losonczy, A. (2016). Distinct contribution of adult-born hippocampal granule cells to context encoding. *Neuron*, *90*(1), 101-112. |
| 21 | Dombeck, D. A., Harvey, C. D., Tian, L., Looger, L. L., & Tank, D. W. (2010). Functional imaging of hippocampal place cells at cellular resolution during virtual navigation. *Nature neuroscience*, *13*(11), 1433. |
| 22 | Busche, M. A., Chen, X., Henning, H. A., Reichwald, J., Staufenbiel, M., Sakmann, B., & Konnerth, A. (2012). Critical role of soluble amyloid-β for early hippocampal hyperactivity in a mouse model of Alzheimer’s disease. *Proceedings of the National Academy of Sciences*, *109*(22), 8740-8745. |
| 27 | Mizrahi, A., Crowley, J. C., Shtoyerman, E., & Katz, L. C. (2004). High-resolution in vivo imaging of hippocampal dendrites and spines. *Journal of Neuroscience*, *24*(13), 3147-3151. |
| 28 | Rickgauer, J. P., Deisseroth, K., & Tank, D. W. (2014). Simultaneous cellular-resolution optical perturbation and imaging of place cell firing fields. *Nature neuroscience*, *17*(12), 1816. |
| 29 | Rajasethupathy, P., Sankaran, S., Marshel, J. H., Kim, C. K., Ferenczi, E., Lee, S. Y., ... & Liston, C. (2015). Projections from neocortex mediate top-down control of memory retrieval. *Nature*, *526*(7575), 653. |
| 30 | Lovett-Barron, M., Kaifosh, P., Kheirbek, M. A., Danielson, N., Zaremba, J. D., Reardon, T. R., ... & Losonczy, A. (2014). Dendritic inhibition in the hippocampus supports fear learning. *Science*, *343*(6173), 857-863. |
| 31 | Sheffield, M. E., & Dombeck, D. A. (2015). Calcium transient prevalence across the dendritic arbour predicts place field properties. *Nature*, *517*(7533), 200. |
| 32 | Gu, L., Kleiber, S., Schmid, L., Nebeling, F., Chamoun, M., Steffen, J., ... & Fuhrmann, M. (2014). Long-term in vivo imaging of dendritic spines in the hippocampus reveals structural plasticity. *Journal of Neuroscience*, *34*(42), 13948-13953. |
| 33 | Kaifosh, P., Lovett-Barron, M., Turi, G. F., Reardon, T. R., & Losonczy, A. (2013). Septo-hippocampal GABAergic signaling across multiple modalities in awake mice. *Nature neuroscience*, *16*(9), 1182. |

**Supplementary Notes**

***Note S1*** *on calibration*

It is important to note that the TM is characterized for spatially-defined input modes. Therefore, any modification of the position of the fiber with respect to the optical component manipulating the excitation light, or any intermediary optical components, will required the TM to be updated. On one hand, this is the main reason why chronic imaging with MMF has not been demonstrated, i.e. repositioning the MMF with sufficient accuracy such that the TM is still valid will require novel engineering strategies. On the other hand, if the fiber remains in the imaging system, the calibration remains valid for days. We tested this directly and measured a decrease of only 5% in the peak intensity after 7 days.

***Note S2*** *on sparse fluorescence labelling*

Because one-photon fluorescence excitation was used, a detectable signal was generated above and below the focus if fluorescent structures were located within the light path. This always occurs with linear excitation and optical sectioning is generally obtained in point-scanning confocal microscopy by positioning a pinhole in the appropriate Fourier intermediate image plane through which only the in-focus light will pass. As the MMF doesn’t perform a Fourier transform of the signal (but instead a transform described by the TM) such physical pinhole strategy cannot be implemented in our system. The fluorescence from out-of-focus objects will therefore cause a background to be present, which can only be minimized by minimizing the number of fluorescence objects in the imaged volume. For this reason, a higher image quality is achieved in sparsely labeled samples.

***Note S3*** *on imaging in freely-moving animals*

Imaging in freely-moving animals with MMF has not yet been carried out as further developments are required to enable it. The primary reason for this to be challenging is that the TM will change as the fiber is deformed during motion of the animal. Fortunately, it has been demonstrated that this change is limited for fiber bending of up to 15% with the 50-μm diameter / NA 0.22 MMF^S1^. It has also been shown that the TM from MMFs with a larger NA incurs smaller changes^S2^. Increasing the NA is thus not only desirable for improving the spatial resolution but also for making the imaging system more suitable for imaging in freely moving animals. Such considerations are only facilitating and the needs to update the TM as a function of deformation remains, i.e. fewer updates will be needed per degree of deformation, but the total deformation can be large. A single solution has been proposed on how to correct the TM: cameras are used to monitor accurately the shape of the fiber and theoretical corrections are applied to the TM^S1^. This would require substantial modification of the imaging algorithm. It would also require a DMD with its fast fresh-rate for wavefront shaping for updating the TM in real-time with the animal movements.

**Supplementary Methods**

*Experimental geometry*

A monochromatic, linearly polarized light beam (λ = 488 nm, CrystalLaser, DL488-020-S) was split and coupled into two separate optical fibers (a polarization maintaining single-mode fiber (PM-SMF, Thorlabs, P1-488PM-FC-2) and a custom SMF (C-SMF, Thorlabs, P1-405B-FC-5), cleaved with a 10º angle for power stability. Light exiting the PM-SMF was collimated (f = 60 mm, achromatic doublet) and its polarization corresponded to the working polarization of the LC-SLM (Meadowlark Optics, HSPDM512, 512 × 512 pixels). The light was phase modulated by the LC-SLM in an off-axis regime and Fourier transformed by a plano-convex (f = 100 mm) lens onto an iris that only transmits the first diffraction order. The transmitted signal was then reflected by a dichroic mirror (Thorlabs, MD498) and circularly polarized by a quarter-wave plate to assure minimum coupling between polarization states. Circularly polarized light then entered a telescope consisting of plano-convex (f = 50 mm) and aspheric (f = 8 mm) lenses coupling the light into a multimode fiber (Thorlabs, FG050UGA, NA = 0.22). The subsequent light path depended on the mode of operation of the optical system. The standard experimental protocol consisted of two modes of operation: acquisition of the TM (system calibration) and image acquisition.

*Acquisition of TM*

In this mode, the light exiting the multimode fiber was coupled into the calibration unit. Here the light output was imaged by a microscope objective lens (Olympus 20×, RMS20X, NA 0.4) and an achromatic doublet (f = 150 mm) onto a CCD camera (Basler pilot, piA640-210gm). In-between the lenses, the signal was converted back into the linear polarization state using a quarter-wave plate and merged with a reference signal using a 50:50 non-polarizing beam-splitter. The TM has a linear relation between bases of input and output modes^S3^. In our implementation, the input modes were diffraction-limited focal points defined across an orthogonal grid (50×50) at the input facet of the MMF and the basis of 120×120 output modes were analogously defined along the plane of the MMF output facet, or any plane axially displaced away from the fiber facet, imaged onto the CCD, thus allowing for volumetric imaging. The LC-SLM was used to sequentially generate input modes that propagate through the MMF and left as a linear combination of output modes, which interfered with the reference signal at the CCD. An image was recorded for different phase steps of each input modes. The output modes were analyzed by a corresponding number (120×120) of CCD camera pixels, thus resulting in one vector of the TM matrix. After acquisition of all input modes the TM measurement was completed and was used to design input fields (and the corresponding LC-SLM modulation) necessary for generation of individual output modes (diffraction-limited foci at the MMF output) or any other desired light field leaving the MMF. Further details of the approach are available elsewhere^S4^.

*Point-scanning-based image acquisition*

Once calibrated, the microscope objective beneath the MMF was exchanged for the sample unit, this houses *ex vivo* neural tissue or the anesthetized animal. Once in position, the fiber was lowered into the area of interest within the tissue. The LC-SLM generated a linear superposition of input modes (modulated wavefront) across the proximal fiber facet. This linear superposition of input modes constructively interfered to produce a single, diffraction limited focal light-point at the desired distance from the distal fiber end. With a maximum LC-SLM refresh rate of 100 Hz, the system could digitally raster-scan a set of 120×120 points or arbitrary access a portion of them across the fluorescent sample, whereby the emitted wavelength is collected and passed back through the fiber. The intensity of the transmitted response signal was registered for each raster scan position by a photo multiplier tube (PMT, Thorlabs, PMM02) and constituted the pixel value in the final acquired image. For the demonstration of increased field-of-view with an MMF having core diameter of 105 μm (Thorlabs, FG105UCA, NA 0.22) 240×240 output modes and 70×70 input modes were used.

*Software*

The software toolbox for control of the fiber-based imaging system has been extensively described elsewhere^S5^ and is available for download from <http://complexphotonics.dundee.ac.uk/>.

*Resolution measurement*

To assess the spatial resolution of the imaging system, a custom-made
USAF-1951 resolution target was imaged with the MMF system. The resolution was defined by separation of objects according to the Rayleigh criterion: drop of 26% in intensity between two objects with respect to the peak intensity. High dynamic range images were generated by combining four images of the excitation point spread function (PSF) recorded with different relative power (OD0, OD1, OD2, and OD3), as described elsewhere^S6^. Line profiles were generated from the high dynamic range images and used to calculate the full width at half maximum (FWHM) of the excitation PSF by performing a Gaussian fit.

*Ex vivo imaging*

All animal work was carried out in accordance with the Animals (Scientific Procedures) Act, 1986 (UK), and under project and personal licenses approved by the Home Office (UK). Organotypic hippocampal brain slices (350 μm) were prepared from male Wistar rats (postnatal day 7). Slices were plated on Millicell inserts (Millipore) and incubated at 34ºC and 5% CO2 for 7-14 days prior to use. Slices were maintained in 1mL of culture medium (50% Minimum Essential Media, 25% heat-inactivated horse serum, 23% Earl’s Balanced Salt Solution, and 2% B-27 with 6.5 g/L added glucose; ThermoFisher Scientific), which was replaced every 2-3 days. Slices were imaged at room temperature in 1 mL of physiological Tyrode’s solution (in mM: 120 NaCl, 2.5 KCl, 30 glucose,
2 CaCl_2_, 1 MgCl_2_, and 25 HEPES, with 2 ascorbic acid and 1 Trolox added to minimize photodynamic damage; Sigma Aldrich; pH = 7.2-7.4). Dentate granule neurons were loaded with fluorescent dye using whole-cell patch electrophysiology. Briefly, glass electrodes (4-8 MΩ resistance) were filled with standard internal solution (in mM: 135 KGluconate, 10 KCl, 10 HEPES, 2 MgCl_2_, 2 Na_2_ATP and 0.4 Na_3_GTP; pH = 7.2-7.4) containing either 2 mM Alexa Fluor 488 fluorescent dye (ThermoFisher Scientific) for structural imaging or 1 mM of the Ca2+ sensitive dye, Oregon Green BAPTA-1 (ThermoFisher Scientific), for functional imaging. Cells were patched for 5-10 minutes to give adequate time for dye diffusion, after which the patch electrode was slowly retracted, enabling the plasma membrane to reseal. Structural and functional images were obtained through an MMF. During functional imaging, 500 μL of a high potassium Tyrode’s solution (in mM: 32.5 NaCl, 90 KCl, 30 glucose, 2 CaCl_2_, 1 MgCl_2_, and 25 HEPES, with 2 ascorbic acid and 1 Trolox added to minimize photodynamic damage; pH = 7.2-7.4) was added to the bath to drive spontaneous neuronal activity. For purposes of comparison, structural images were additionally acquired using a confocal microscope (BioRad Radiance 2000) equipped with a 488 nm argon laser line and controlled by LaserSharp (BioRad) software. Confocal images were acquired as a high resolution (1024 x 1024 pixels) z-stack (1 μm step size), taken through a 60× water-immersion objective (0.9 NA; Olympus) on an upright Olympus BX50WI microscope.

*In vivo imaging*

All experiments were approved by the local ethical review committee at the University of Oxford and licensed by the UK Home Office. Transgenic Thy1-GFP-M mice (line 007788, Jackson Laboratories) expressing eGFP in sparse subsets of neurons were used for *in vivo* imaging of dendritic spines. Animals aged 4–6 weeks were premedicated with intraperitoneal injections of dexamethasone (Dexadreson, 4 μg), atropine (Atrocare, 1 μg) and carprofen (Rimadyl, 0.15 μg). General anesthesia was induced by an intraperitoneal injection of fentanyl (Sublimaze, 0.05 mg/kg), midazolam (Hypnovel, 5 mg/kg), and medetomidine (Domitor, 0.5 mg/kg). Mice were then placed in a stereotaxic frame equipped with mouth and ear bars. Depth of anesthesia was monitored by pinching the rear foot and by observation of the respiratory pattern. Body temperature was closely monitored throughout the procedure and kept constant at 37ºC by the use of a heating mat and a temperature controller in conjunction with a rectal temperature probe. Both eyes were covered with eye ointment (Maxitrol, Alcon) to prevent corneal desiccation during the experiment. The skin over the craniotomy site was shaved and an incision was made to expose the skull, after which a hole of 0.5 mm diameter was drilled (Foredom K.1070, Blackstone Industries, CT, USA) into the skull with a
0.4 mm drill bit. The craniotomy was centered at 1.3 mm anterior and 1.0 mm lateral to bregma. Cyanoacrylate glue (Pattex Classic, Henkel, Germany) was applied to the surrounding skull, muscle, and wound margins to prevent further bleeding. A small metal bar was attached to the skull over the left hemisphere with dental cement, which was also used to cover all exposed areas of skull. The mouse was then placed on a custom-made stage, its head fixed to the stage using the steel bar, for imaging. Subsequently, the multimode optic fiber (MMF, total diameter 125 μm) was gradually lowered up to 1.8 mm into the brain tissue, targeting the dorsal striatum.

For dynamic imaging, C57BL/6 mice were injected with very small amounts (<5 nL) of a 1:1 mixture of highly diluted (1:50000-100000 in PBS) AAV1.hSyn.Cre.WPRE.hGH (Penn Vector Core) and AAV1.CAG.Flex.GCaMP6m.WPRE.bGH (Penn Vector Core) into the right medial geniculate body of the thalamus. The thalamic stereotaxic coordinates were 2.9 mm posterior to bregma, 2.05 mm to the right of the midline and 3.0 mm from the cortical surface. Fiber-imaging was performed 3-4 weeks after GCaMP6m viral injections. The pre-imaging surgery was as described above, but with a different anesthetic regime: general anesthesia was induced with ketamine (100 mg/kg, Vetalar) and medetomidine (140 μg/kg), and ketamine (50 mg/kg/h) and medetomidine (0.07 mg/kg/h) were regularly topped up at 30 min intervals to maintain a stable level of anesthesia throughout the experiment. Sound presentation was performed through a free-field loudspeaker (Tucker-Davis Technologies) placed near the ear canal of the mouse’s left ear. Stimuli were single 16 kHz pure tones of 100 milliseconds duration.

At the end of the imaging session, the mouse was given an overdose of sodium pentobarbital
(240 mg/kg) prior to transcardial perfusion with phosphate buffered saline (PBS) and then 4% paraformaldehyde. The fixed brain was then extracted and section to confirm the location of the fiber tract.

**Supplementary References**

S1. Plöschner, M., Tyc, T. & Čižmár, T. Seeing through chaos in multimode fibres. *Nat. Photonics* **9,** 529–535 (2015).

S2. Loterie, D., Psaltis, D. & Moser, C. Bend translation in multimode fiber imaging. *Opt. Express* **25,** 6263 (2017).

S3. Popoff, S. M. *et al.* Measuring the Transmission Matrix in Optics: An Approach to the Study and Control of Light Propagation in Disordered Media. *Phys. Rev. Lett.* **104,** 100601 (2010).

S4. Plöschner, M. & Čižmár, T. Compact multimode fiber beam-shaping system based on GPU accelerated digital holography. *Opt. Lett.* **40,** 197 (2015).

S5. Plöschner, M., Straka, B., Dholakia, K. & Čižmár, T. GPU accelerated toolbox for real-time beam-shaping in multimode fibres. *Opt. Express* **22,** 2933 (2014).

S6. Ohayon, S., Caravaca-Aguirre, A., Piestun, R. & DiCarlo, J. J. Minimally invasive multimode optical fiber microendoscope for deep brain fluorescence imaging. *Biomed. Opt. Express* **9,** 1492 (2018).
